# Ellagitannin, Phenols, and Flavonoids as Antibacterials from *Acalypha arvensis* (Euphorbiaceae)

**DOI:** 10.3390/plants11030300

**Published:** 2022-01-24

**Authors:** Ever A. Ble-González, Abraham Gómez-Rivera, Alejandro Zamilpa, Ricardo López-Rodríguez, Carlos Ernesto Lobato-García, Patricia Álvarez-Fitz, Ana Silvia Gutierrez-Roman, Ma Dolores Perez-García, Alejandro Bugarin, Manasés González-Cortazar

**Affiliations:** 1 División Académica de Ciencias Básicas, Universidad Juárez Autónoma de Tabasco, Carretera Cunduacán-Jalpa Km. 0.5, Cunduacán 86690, Tabasco, Mexico; abgori@gmail.com (A.G.-R.); ricardo.lopezr@ujat.mx (R.L.-R.); carlos.lobato@ujat.mx (C.E.L.-G.); 2 Centro de Investigación Biomédica del Sur, Instituto Mexicano del Seguro Social, Argentina No. 1, Col. Centro, Xochitepec 62790, Morelos, Mexico; azamilpa_2000@yahoo.com.mx (A.Z.); 12gtr.ana@gmail.com (A.S.G.-R.); lola_as@yahoo.com.mx (M.D.P.-G.); 3 Laboratorio de Toxicología, Cátedra CONACyT-Universidad Autónoma de Guerrero, Av. Lázaro Cárdenas s/n. Col. La Haciendita, Chilpancingo 39070, Guerrero, Mexico; paty_fitz@hotmail.com; 4 Department of Chemistry and Physics, Florida Gulf Coast University, Fort Myers, FL 33965, USA; abugarin@fgcu.edu

**Keywords:** *Acalypha arvensis*, ellagitannin, corilagin, *Staphylococcus aureus*, flavonoids, antibacterial

## Abstract

There is a significant need to gain access to new and better antibacterial agents. *Acalypha arvensis,* a plant from the Euphorbiaceae family, has been used in traditional medicine for centuries to treat infectious diseases. This manuscript reports the isolation, characterization, and antibacterial screening of 8 natural products extracted from maceration of aerial parts of *Acalypha arvensis.* Specifically, three extracts were assessed (*n*-hexane, ethyl acetate, and ethanol), in which antibacterial activity was evaluated against diverse bacterial strains. The ethanolic extract showed the best activity against methicillin-sensitive and methicillin-resistant *Staphylococcus aureus*, *Klebsiella pneumoniae,* and *Pseudomonas aeruginosa* strains, which supports the medicinal properties attributed to this plant. The chromatographic fractions AaR4 and AaR5 were the most bioactive, in which the ellagitannin natural product known as corilagin (**1**) was identified for the first time in this plant. Therefore, it can be said that this is the main chemical responsible for the observed antibacterial activity. However, we also identified chlorogenic acid (**2**), rutin (**3**), quercetin-3-*O*-glucoside (**4**), caffeic acid (**5**), among others (**6**–**8**). Hence, this plant can be considered to be a good alternative to treat health-related issues caused by various bacteria.

## 1. Introduction

The World Health Organization (WHO) has reported that microbial diseases (both from bacteria and fungi) are a leading cause of death in humans [1], which can be transmitted directly or indirectly from one individual to another, and that bacterial resistance causes more than 10 million deaths per year [2,3,4,5]. The microorganisms of greatest interest that cause infectious diseases in humans are bacteria such as *Salmonella typhi* (causing typhoid fever), *Staphylococcus aureus* (causing skin infections, sometimes pneumonia, endocarditis, and osteomyelitis), *Streptococcus pneumoniae* (causing pneumonia), among others [6,7]. The cosmopolitan increase of bacterial infections caused mainly by the inappropriate use of antibiotics and/or a deficient control of infections has led to the rise of drug-resistant strains, which represent a major threat to public health and the global economy [1]. Therefore, seeking and development of new generations of antimicrobials to mitigate the spread of antibiotic resistance have become imperative [8]. In fact, a reexamination of traditional medicines has become more common among scientists. This approach has already produced new antibiotics, and it is expected that more novel antibiotics will be discovered/isolated from traditional medicinal plants in the future. [9] Those new bioactive molecules from plant extracts could be essential secondary metabolites that will aid further understand antimicrobial activity, while advancing drug discovery [10].

It is well known that the medicinal properties of plants are related to their ability to synthesize a wide range of bioactive compounds. One of the main families of bioactive molecules found in plant extracts is the phenolic family. These compounds have shown excellent antibacterial activity against resistant pathogens through various reported mechanisms [10,11,12] and therefore are an important group of potential medicines.

The species *Acalypha arvensis* Poepp Endl., usually known as “spider leaf”, “couch grass”, “borreguillo”, and/or “worm weed”, belongs to the family Euphorbiaceae and it can be found from Mexico to tropical South America [13,14]. In traditional medicine, this plant has been used to treat illnesses such as diarrhea, vomiting, scabies, mouth sores (canker sores), spider bites, snake bites, cancer, athlete′s foot, inflammation, fluid retention, headache, and wound healing, among others [14,15,16,17]. One of the few pharmacological studies carried out on *A. arvensis* reported that an ethanol extract from leaves and flowers presented antimicrobial activity against enterobacteria pathogenic to humans; *Salmonella typhi* was the most inhibited bacterium (33.73%), whereas *Escherichia coli* (7.35%) was the most resistant [18]. The ethanol extract was also evaluated against three Gram-positive bacteria that cause respiratory infections (*Staphylococcus aureus, Streptococcus pneumoniae and Streptococcus pyogenes*), showing activity only against *Staphylococcus aureus* [19]. In another study, conducted on five species of the genus *Acalypha*, biocidal activity was evaluated, and it was demonstrated that *A. arvensis* has activity against *Pseudomonas aeruginosa* and *Cryptococcus neoformans* at a concentration of 1 mg/mL. On the other hand, it did not show activity against mosquito larvae (*Aedes aegypti* and *Anophles albinamus*). Groups of compounds such as flavonoids, anthocyanins, anthraquinones, coumarins, saponins, and cardenolides were identified in those extracts [20]. However, there are no prior reports indicating the actual bioactive molecules present in this plant species. Thus, the main aims of this work were (1) to evaluate the antimicrobial activity of three extracts and four fractions from *A. arvensis* against microorganisms sensitive and resistant to methicillin and (2) to determine the actual secondary metabolites responsible for the observed bioactivity.

## 2. Results

### 2.1. Antibacterial Effect of A. arvensis’s Extracts

Maceration of the aerial parts of *Acalypha arvensis* (Aa) species provided the following extracts: hexane (AaHex, 7.4 g, 0.76%), ethyl acetate (AaAcOEt, 19.2 g, 1.98%), and ethanol (AaEtOH, 11.24 g, 1.15%). The three extracts were evaluated against 13 strains of sensitive and methicillin-resistant bacteria (Table 1).

According to the results in Table 1, the extract that showed the best antibacterial activity in relation to the 13 strains of bacteria evaluated was the ethanolic extract (AaEtOH) with good activity against four strains of Gram-positive bacteria (Sa, SaRM1, SaRM2, and Se) and three strains of Gram-negative bacteria (Kp1, Kp2, and Pa), followed by the ethyl acetate extract (AaAcOEt) that only showed activity against *S. aureus* (Sa) and *P. aeroginosa* (Pa). Lastly, the hexane extract (AaHex) did not exhibit any relevant activity against any of the strains evaluated at the screened concentration. Therefore, the AaEtOH extract was selected for chromatographic fractionation and to carry out the bacterial evaluation.

### 2.2. Antibacterial Activity of the AaEtOH Fractions (AaR1, AaR2, AaR3, AaR4, and AaR5)

Chromatographic fractionation of the AaEtOH extract produced five clusters, of which only four were evaluated (since AaR1 offered very low yield), against sensitive *S. aureus* (Sa) and methicillin-resistant *S. aureus* (SaRM1) (Table 2).

Table 2 shows the significant antibacterial activity from fractions AaR4 and AaR5 against both *S. aureus* sensitive (Sa) and methicillin-resistant (SaRM1) strains.

### 2.3. HPLC Analysis of Extract and Fractions

**Figure 1:**
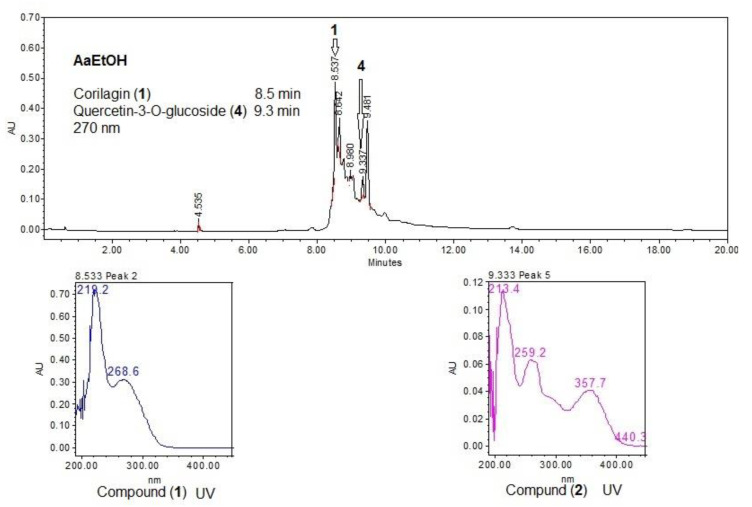
HPLC chromatogram of the AaEtOH extract and UV spectra of compounds (**1**) and (**4**).

Analysis of the AaEtOH extract by high-performance liquid chromatography (HPLC) allowed the identification of four main compounds that have retention times of 8.5, 8.6, 9.3, and 9.4 min and UV-Vis spectra of (λ_max_ = 191.1, 219, 258, 268, and 355 nm), respectively. Those values could correspond to groups of phenolic-type compounds (Figure 1).

**Figure 2:**
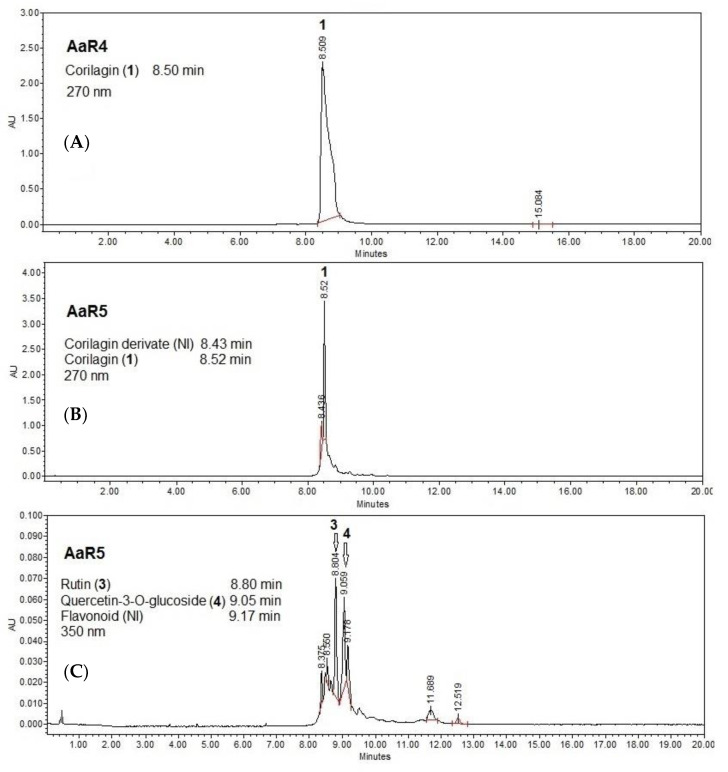
HPLC chromatograms of the fractions: (**A**) AaR4, (**B**) AaR5 at 270 nm and (**C**) AaR5 at 350 nm. Corilagin (**1**), corilagin derivative (Unidentified, NI), rutin (**3**), quercetin-3-O-glucoside (**4**) and caffeic acid (**5**) are identified.

Figure 2A,B show the HPLC chemical analysis of two active fractions (AaF4 and AaF5) at 270 nm and Figure 2C shows fraction AaF5 at 350 nm.

In the chromatograms of Figure 2, it can be observed that compound **1** and other derivatives of the same type (ellagitannins) that could not be identified (NI) are present in the two fractions. Therefore, it could be said that the antimicrobial effect produced by the two fractions (AaR4 and AaR5) could be mainly due to the ellagitannin known as corilagin (**1**) and the presence of flavonoids. According to HPLC, the concentration of compound **1** (8.5 min, 270 nm) in the ethanol extract is 18.36 mg of corilagin/g of extract.

### 2.4. Isolation and Structural Elucidation of Compounds ***1**–**3***

Although both fractions (AaR4 and AaR5) were active, it was decided to isolate the compounds from fraction AaR4 since it provided a higher yield.

**Figure 3:**
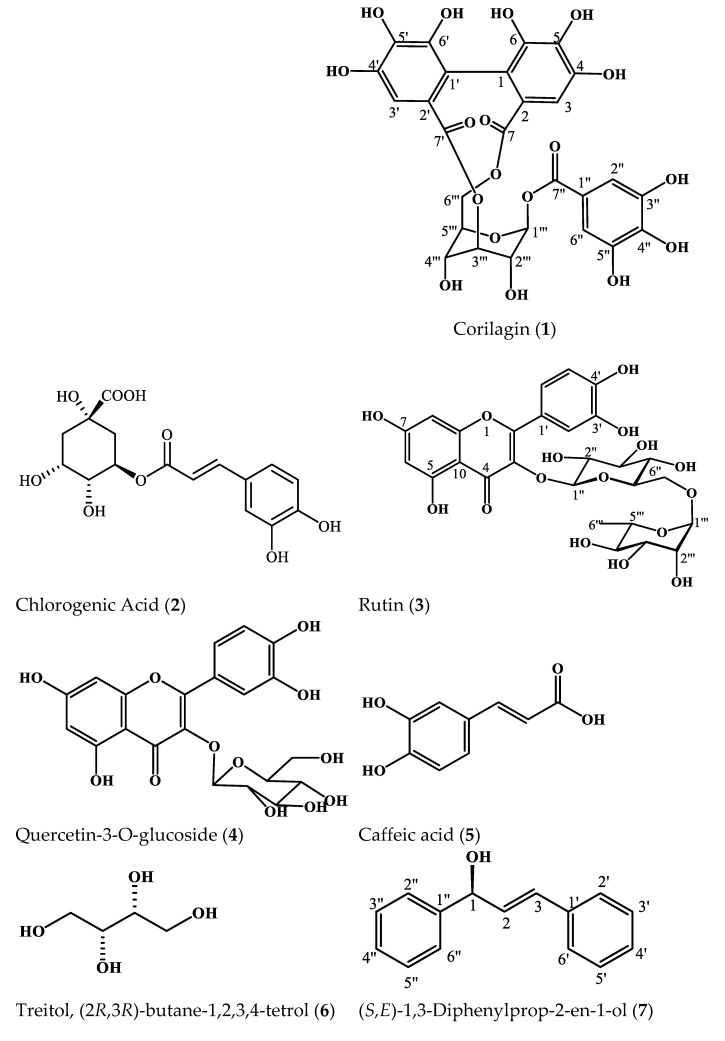

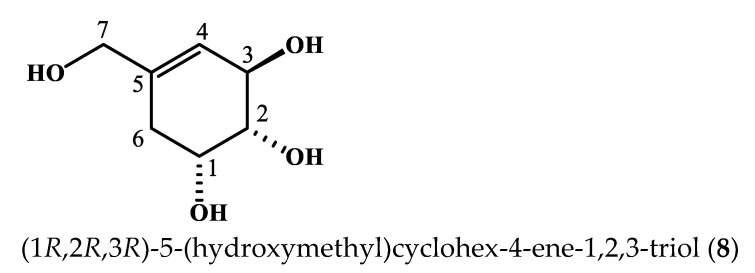
Chemical structures of the compounds isolated from *Acalypha arvensis (***1–8**) in the ethanol extract (AaEtOH).

Chromatographic fractionation of the AaR4 fraction yielded seven compounds. Compound **1** was obtained as an amorphous yellow precipitate. The HPLC chromatogram and UV absorption spectrum (Figure S1) showed a retention time of 8.5 min with absorption lengths of λ_max_ = 221 and 268 nm. Analysis of the ^1^H NMR spectrum showed three characteristic signals for three aromatic rings at δ 7.06 (2H, s, H-2′′ and H-6′′), 6.69 (1H, s, H-3′) and 6.67 (1H, s, H-3), assigned to gallic acid. Additionally, a doublet signal is observed at δ 6.36 (1H, d, *J* = 2.2 Hz, H-1′′′′) and its ^13^C-NMR chemical shift at δ 95.0 (CH, C-1′′′′) corresponds to an anomeric carbon of a sugar. The COSY experiment analysis allowed us to identify by proton couplings a hexose named α-D-glucose. The anomeric proton (δ 6.36) shows long range correlation (HMBC) with the signal of the carbonyl at δ 166.6 assigned to C-7′′ of the gallic acid, so this acid is substituted at C-1′′′ position of glucopyranose. Likewise, it is observed a coupling to protons H-6′′′′a and H-6′′′′b (δ 4.15 and 4.96, respectively) with another carbonyl at δ 170.0. Thus, this was assigned to C-7 of another gallic acid, also for H-3′′′′ (δ 4.8) with the carbonyl signal at δ 168.5 from C-7′ of the third gallic acid. Analysis of the obtained one-dimensional (^1^H, ^13^C, and DEPT) and two-dimensional (COSY, HMQC, and HMBC) NMR spectra ( supplementary data Figures S2–S7), and comparison with data described in the literature [21,22,23], this compound was identified as an ellagitannin (β-1-*O*-galloyl-3,6-(R)-hexahydroxydiphenoyl-α-D-glucose) known as corilagin (**1**, Figure 3).

**Figure 4:**
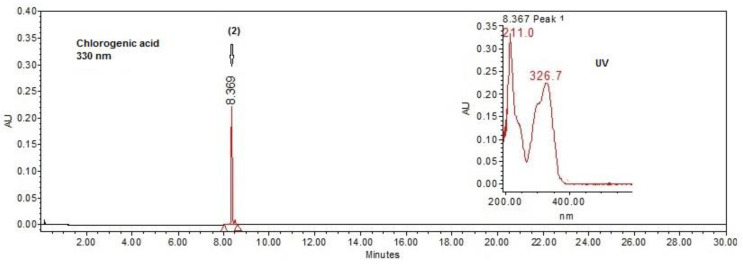
HPLC chromatogram of chlorogenic acid (**2**) and its UV spectrum.

**Figure 5:**
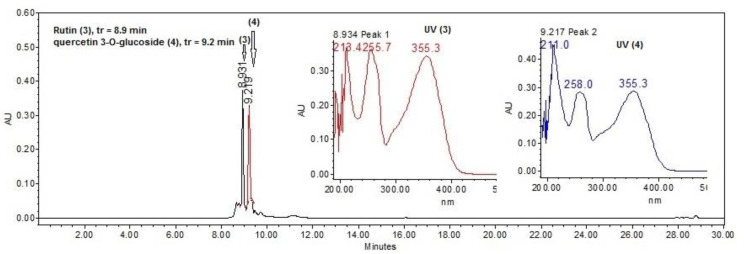
HPLC chromatogram of rutin (**3**), quercetin 3-O-glucoside (**4**), and their UV spectra.

**Figure 6:**
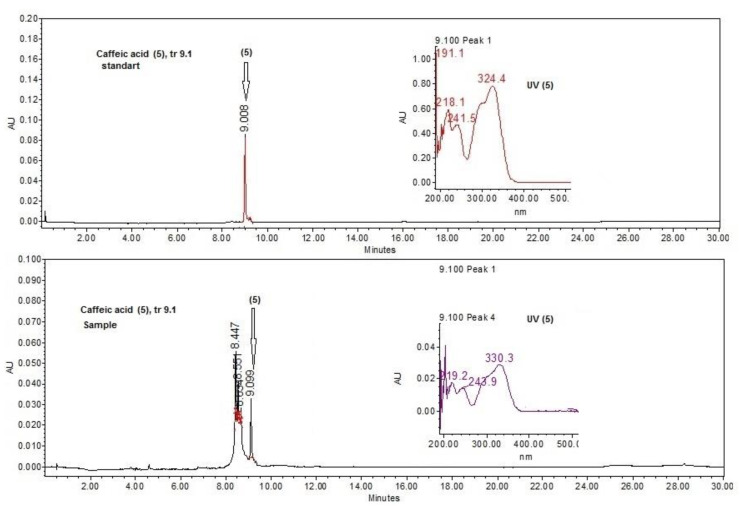
HPLC chromatograms of caffeic acid (**5**) compared to the standard and its UV spectrum.

Additional analysis of fraction AaR4 was further separated using a second column chromatography. This second separation delivered 3 fractions (fractions 8–10, 13–20, and 23–27). Analysis of these fractions by HPLC and UV-Vis spectra indicated that peaks at 8.36 min (λ_max_ = 211 and 326 nm) co-respond with chlorogenic acid (**2**, Figure 4), 8.9 min (λ_max_ = 213, 255.7, and 355.3 nm) with rutin (**3**, Figure 5), 9.2 min (λ_max_ = 213.4, 255.7 and 355.3 nm) with quercetin-3-*O*-glucoside (**4**, Figure 5), and 9.1 min (λ_max_ = 219.2, 243.9 and 330.3 nm) with caffeic acid (**5**, Figure 6), respectively. These were compared with known standards to stablish their identity.

Fraction 32–37 was analyzed by 1D and 2D NMR spectroscopy (Figures S8–S13) and by comparison with spectroscopic data from the literature [24,25,26,27], it was found a mixture of a polyol known as treitol (**6**), a dihydrochalcone known as (*S,E*)-1,3-diphenylprop-2-en-1-ol (**7**), and a shikimic acid derivative (1*R*,2*R*,3*R*)-5-(hydroxymethyl)cyclohex-4-ene-1,2,3-triol (**8**) (Figure 3).

Corilagin (**1**): ^1^H NMR (CD_3_OD, 600 MHz) δ 7.06 (2H, s, H-2′′ y H-6′′), 6.69 (1H, s, H-3′), 6.67 (1H, s, H-3), 6.36 (1H, d, *J* = 2.2 Hz, H-1′′′), 3.99 (1H, dd, 2.0, 3.5 Hz, H-2′′′), 4.8 (1H, m, H-3′′′), 4.46 (1H, dd, *J* = 1.7, 3.3 Hz, H-4′′′), 4.52 (1H, dd, *J* = 8.0, 10.9 Hz, H-5′′′), 4.15 (1H, dd, *J* = 8.0, 10.9 Hz, H-6a′′′), 4.96 (1H, dd, *J* = 10.9, 10.9 Hz, H-6b′′′); ^13^C NMR (CD_3_OD, 150 MHz) δ 62.4 (CH, C-4′′′), 64.9 (CH_2_, C-6′′′), 69.4 (CH, C-2′′′), 71.5 (CH, C-3′′′), 76.1 (CH, C-5′′′), 95.0 (CH, C-1′′′), 108.3 (CH, C-3), 110.2 (CH, C-3′), 110.9 (2CH, C-2′′ y C-6′′), 116.6 (C, C-1), 117.2 (C, C-1′), 120.7 (C, C-1′′), 125.4 (C, C-2), 125.5 (C, C-2′), 137.6 (C, C-5), 138.1 (C, C-5′), 140.3 (C, C-4′′), 145.1 (C, C-6′), 145.2 (C, C-6), 145.6 (C, C-4′), 146.0 (C, C-4), 146.3 (2C, C-3′′ y C-5′′), 166.6 (C, C-7′′), 168.5 (C, C-7′), 170.0 (C, C-7).

Treitol *o* (2*R*,3*R*)-butane-1,2,3,4-tetrol (**6**)**:** ^1^H NMR (CD_3_OD, 600 MHz) δ 3.66 (2H, dd, *J* = 5.7, 5.9 Hz, H-2, H-3), 3.52 (2H, dd, *J* = 5.9, 11.2 Hz, H-1a, H-4a), 3.60 (2H, dd, *J* = 4.9, 11.2 Hz, H-1b, H-4b); ^13^C NMR (CD_3_OD, 150 MHz) δ 73.7 (CH, C-2 y C-3), 64.3 (CH_2_, C-1 y C-4).

(*S*,*E*)-1,3-Diphenylprop-2-en-1-ol (**7**): ^1^H NMR (CD_3_OD, 600 MHz) δ 5.31 (1H, d, *J* = 6.6 Hz, H-1), 6.39 (1H, dd, *J* = 6.6, 15.8 Hz, H-2), 6.64 (1H, d, *J* = 15.8 Hz, H-3), 7.42 (1H, dd, *J*= 1.3, 8.4 Hz, H-2′′ y H-6′′), 7.39 (dd, *J* = 1.3, 8.4 Hz, H-2′ y H-6′), 7.20 (dd, *J* = 7.4, 7.4 Hz, H-3′ y H-5′), 7.26 (dd, *J* = 7.4, 7,4 Hz, H-3′′ y H-5′′), 7.35 (dd, *J* = 7.5, 7,6 Hz, H-4′), 7.28 (dd, *J* = 7.6, 7.9 Hz, H-4′′); ^13^C NMR (CD_3_OD, 150 MHz) δ 75.8 (CH, C-1), 133.3 (CH, C-2), 131.1 (CH, C-3), 127.4 (4CH, C-2′,C-2′′, C-6′ y C-6′′), 128.3 (2CH, C-3′y C-5′), 128.5 (2CH, C-3′′y C-5′′), 129.5 (CH, C-6′′), 129.3 (CH, C-6′), 141.8 (C, C-1′′), 138.2 (C, C-1′).

(1*R*,2*R*,3*R*)-5-(hydroxymethyl)cyclohex-4-ene-1,2,3-triol (**8**)**:** ^1^H NMR (CD_3_OD, 600 MHz) δ 6.78 (1H, d, 3.3 Hz, H-4), 4.37 (1H, dd, 3.6, 4.2 Hz, H-3), 3.67 (1H, m, H-2), 3.98 (1H, dd, 5.4, 7.5 Hz), 2.72 (1H, dd, 4.9, 18.0, H-6a), 2.19 (1H, dd, 5.9, 18.0, H-6b), 3.74 (1H, dd, 2.7, 11.0 Hz, H-7a), 3.60 (1H, m, H-7b): ^13^C NMR (CD_3_OD, 150 MHz) δ 138.2 (CH, H-4), 67.37 (CH, C-3), 72.9 (CH, C-2), 68.3 (CH, C-1), 31.8 (CH_2_, C-6), 131.1 (C, C-5), 64.6 (CH_2_, C-7).

## 3. Discussion

Considering the current uses in traditional medicine and the antibacterial activity reports from the title plant, the aim of this work was to evaluate the antibacterial properties of *Acalypha arvensis* and the identification of molecules responsible for the activity.

The emergence and dissemination of methicillin-resistant *Staphylococcus* (MRS) strains is a worrying problem in public health. Therefore, new anti-MRS agents are urgently needed. Species of the genus *Acalypha* have shown antibacterial activity including *A. alnifolia, A. alopecuroidea, A. arvenis, A. fimbriata, A. gaumeri, A. hispida, A. indica, A. monos-tachya, A. platyphilla, A. racemose, A. wilkesiana,* and *A. torta* [28]. In this work, it was demonstrated that the ethanolic extract of *A. arvensis* exerts antibacterial effects against methicillin-sensitive and methicillin-resistant *Staphylococcus aureus, Klebsiella pneumoniae,* and *Pseudomonas aeruginosa* strains, which is consistent with what has been described in the literature, where its effect against strains of *Staphylococcus aureus, Salmonella typhi, Shigella flexneri, Mycobacterium intracellurare, Thychophyton mentagrophyte,* and *Saccharomyces cerevisiae* has been reported [18,19,29,30]. There are also reports of antimicrobial potential in other species of the same genus such as *A. diversifolia* where hexane, dichloromethane, and methanol extracts were tested against *Staphylococcus aureus* (ATCC 6538), *Bacillus subtilis* (ATCC 21556), *Klebsiella pneumonia* (ATCC 10031), and *Escherichia coli* (ATCC 9637) and were bioactive [31]. It has also been mentioned that methanolic extract of *Acalypha fruticosa* exhibit positive effects against *Staphylococcus aureus, Bacillus subtilis, Myotis flavus,* and *Staphylococcus epidermis*, while the aqueous extract shows activity against *Streptococcus pyogene, Staphylococcus epidermis, Proteus vulgaris,* and *Escherichia coli* [28].

The activity of AaEOH from *A. arvensis* could be due to phenolic acids and flavonoids that were identified in this extract. In general, tannins, flavonoids, coumarins, saponins, alkaloids, terpenes, coumarins, anthocyanins, and anthraquinones have been previously described in species of this genus [28]. In this study, corilagin (**1**), an ellagitannin, was isolated and identified; this compound has been isolated from different species as well, including those of the genus Euphorbiaceae, such as *Phyllantus niruri* [32]. Some pharmacological activities of corilagin have already been described, such as antiatherogenic [33], antioxidant [34], hepatoprotective [35], antitumor [36], and antibacterial [37]. Corilagin has been reported to exhibit antimicrobial activity due to its inhibitory effect against *S. aureus, E. coli, P. aeruginosa,* and *K. pneumoniae* with a MIC of 1024 pg/mL [38]. Tannins have antibacterial effects, inhibiting the growth of Gram-positive and Gram-negative bacteria, and most compounds have bacteriostatic properties. The MICs of several tannins range from 61.5 to 3200 µg/mL [39]. Some of the proposed mechanisms of how these compounds (gallotannins) act involve their iron chelating property. Iron is essential for optimal bacterial growth. Siderophores, low-molecular-weight organic compounds produced by bacteria, can solubilize iron in the external environment and make it available to bacteria. Gallotannins can chelate ferric iron from their environment, making iron unavailable to bacteria, leading to inhibition of bacterial growth due to iron deprivation. Furthermore, the iron chelation efficiency of gallotannins is correlated with the number of galloyl groups, with increasing degrees of galloylation reducing the iron-binding capacity due to steric effects. It has also been shown that tannins can inhibit bacterial cell-wall synthesis by inactivating the enzymes involved or by direct binding [40]. On the other hand, chlorogenic acid has been shown to have broad-spectrum antibacterial activity and some inhibition against *E. coli* and *S. aureus*. Reports indicate that the antibacterial mechanism of chlorogenic acid may be related to noncompetitive inhibition of arylamine acetyltransferase in bacteria, as well as changing the permeability of cell membranes by inhibiting a change in β-galactosidase. This reduces the concentration of sugar and acetone in the metabolic process of bacteria and hindering the metabolism and protein synthesis of the strain, resulting in insufficient energy and further affecting the bacteria’s growth and reproduction [41].

It has been reported that plants rich in phenolic acids and flavonoids, two types of metabolites present in the ethanolic extract of *A. arvensis*, possess a broad spectrum of antimicrobial activity [42]. In particular, the amount and position of the hydroxyl groups in these types of compounds, such as rutin, quercetin glycoside, and caffeic acid present in *A. arvensis*, have been shown to be related to their antibacterial effect, mainly against *S. aureus.* The presence of these functional groups affects lipophilicity, damaging the phospholipids and proteins, resulting in an increase in cell permeability [43,44].

Since there are numerous toxic plants, it is important to evaluate their preclinical toxicological and pharmaceutical action before considering using them for a safe/beneficial treatment and therefore validating them as medicinal plants [45]. As such, it is in our interest to continue with further evaluation (in vivo) of the *Acalypha arvensis* extracts against biological models. Thus far, it has been reported that studies carried out with corilagin revealed that the applied therapeutic dose does not exert adverse effects on the liver [36]. Additionally, the compounds quercetin, gallic acid, corilagin, and ellagic acid protect against some cytotoxic effects of acetaminophen, microcystins, galactosamine, and lipopolysaccharides [46]. Therefore, isolation and chromatographic analysis of polyphenolic compounds such as flavonoids, hydroxycinnamic acid derivatives, and organic acids from plants aid in the understanding of their inherited bioactivity [47,48,49]. In the case of *A. arvensis* extracts, it was found that around (8–10 min) ellagitannin was found during chromatographic analysis. However, there were additional natural products that overlapped at that same retention time. This low resolution could be improved using micro-pillar matrix columns, which have proved to have better separation performance in HPLC [50].

## 4. Materials and Methods

### 4.1. Equipment and Reagents

NMR spectra were recorded on an Agilent DD2-600 at 600 MHz for 1H and 150 MHz for ^13^C NMR, using CD_3_OD as the solvent. Chemical shifts are reported in ppm relative to TMS. Thin-layer chromatography (TLC) was performed using TLC Silica gel 60, F254, and 20 × 20 cm aluminum sheets (Merck KGaA, Darmstadt, Germany). High-performance liquid chromatography (HPLC, Milford, MA, USA) analyses were performed on a Waters 2695 Separation module system, equipped with a photodiode array detector (Waters Co. 2996) and Empower 3 software (Waters Corporation, Milford, MA, USA).

### 4.2. Plant Material

The aerial parts of *Acalypha arvensis* were collected in Pechucalco 1st section of the municipality of Cunduacán, Tabasco, Mexico; during the month of April 2021. One specimen was deposited in the Herbarium of the Academic Division of Biological Sciences of the Universidad Juárez Autónoma de Tabasco for taxonomic identification and safekeeping (Voucher No. 036228). The fresh plant material of *Acalypha arvensis* was dried at room temperature, under shade for 72 h.

### 4.3. Extracts

Dried material (820 g) was milled in a grinder (Pulvex, particle size 4 mm). The extraction process was through a serial maceration with solvents of ascending polarity (*n*-hexane, ethyl acetate and ethanol). This to extract all the secondary metabolites according to their dissolution affinity. Initially, *n*-hexane (2.0 L, Merck) was added to the dry material and allowed to stand for 24 h at room temperature (25–30 °C). Subsequently, it was filtered (Whatman No. 4 paper) and concentrated in a rotary evaporator (Heidolph G3, Germany) under reduced pressure, to obtain the hexane extract (AaHex, 7.4 g). This process was repeated in triplicate. The same plant material dried after extraction with hexane was macerated with ethyl acetate (2.0 L, Merck, Mexico City, Mexico). The same procedure mentioned above was followed, to give the ethyl acetate extract (AaAcOEt, 19.2 g). Finally, the ethanolic (AaEtOH, 11.2 g) was obtained following the same protocol. All extracts were lyophilized (Heto Drywinner DW3) and tested by the biological model used for this study.

### 4.4. Isolation and Identification of Compounds *(**1**–**8**)*

**Scheme 1:**
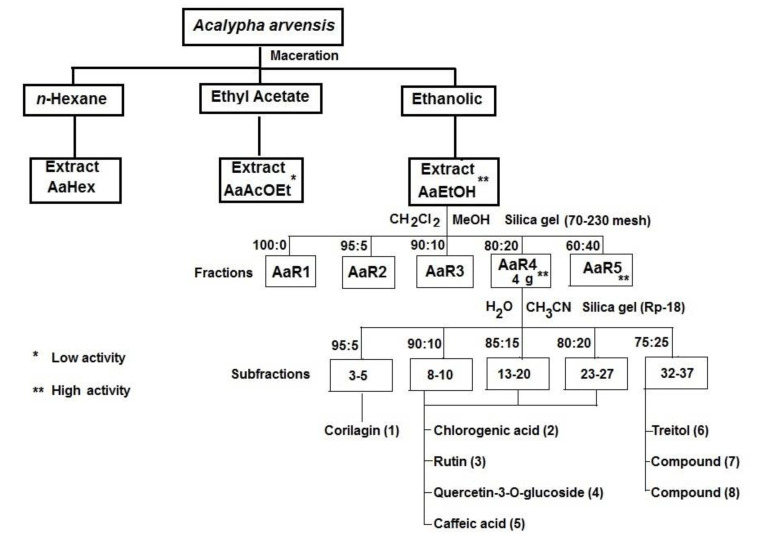
Protocol used for the isolation of active compounds from *Acalypha arvensis*.

Ethanolic extract (AaEtOH, 11.2 g) was adsorbed with silica gel (60 g, gel 60, Merck) and fractionated in a glass column (600 × 50 mm) packed with silica gel (100 g, 70–230 mesh, Merck) as stationary phase. Dichloromethane was used as the mobile phase with gradual increase of polarity using 10% *v*/*v* methanol, collecting 32 fractions of 200 mL. All samples were concentrated under reduce pressure using a rotary evaporator (Heidolph Laborota 4000) and lyophilized. Chromatographic analysis by CCF allowed the assemblies in five fractions: AaR1 (0.05 g), AaR2 (1.5 g), AaR3 (2.2), AaR4 (4.2 g), and AaR5 (1.8 g). Fraction AaR4 (4 g, column 2) was adsorbed on silica gel (7 g, Rp-18, Merck) as a stationary phase and water with a decrease in polarity with acetonitrile at 5% *v*/*v* was used as a mobile phase, collecting 50 fractions of 30 mL each. A dark yellow precipitate was obtained in fractions 3–5, which was identified as corilagin (**1**). In fractions 8–10, 13–20, and 23–27, chlorogenic acid (**2**), rutin (**3**), quercetin-3-*O*-glucoside (**4**) and caffeic acid (**5**) were identified using HPLC by comparison with commercial standards. In fraction 32–37, a mixture of a polyol called treitol (**6**), a dihydrochalcone known as (*S,E*)-1,3-diphenylprop-2-en-1-ol (**7**) and a shikimic acid derivative (1*R*,2*R*,3*R*)-5-(hydroxymethyl)cyclohex-4-ene-1,2,3-triol (**8**) were identified (see Scheme 1).

### 4.5. Antibacterial Activity

#### 4.5.1. Strains Used

The 13 ATCC bacterial strains used were: Gram-positive; *Staphylococcus aureus* 29213(Sa), methicillin-resistant *Staphylococcus aureus* 43300(SaRM1) and 3359(SaRM2), *Staphylococcus epidermis* 1042(Se), *Staphylococcus haemolyticus* 1165(Sh), and *Enterococcus faecalis* 29212(Ef), Gram-negative; *Klebsiella pneumoniae* 13883(Kp1) and 700605(Kp2), *Pseudomonas aeruginosa* 27853(Pa), *Escherichia coli* 25922(Ec1), 1047(Ec2), and 4036(Ec3) and *Salmonella dublin* 9676(Sd). The strains were maintained on Trypticase Soy Agar (Merck) at 37 °C, 24 h.

#### 4.5.2. In Vitro Evaluation of the Organic Extracts Using a Plate Dilution Method

The antibacterial activity was measured by determining the minimal inhibitory concentration (MIC) and it was carried out using the standard agar dilution method [51]. Briefly, the AaHex, AaAcOEt, and AaEtOH extracts and AaR2, AaR3, AaR4, and AaR5 fractions were dissolved in dimethyl sulfoxide (DMSO; 2% *v*/*v*) and sterile water (8% *v*/*v*); to obtain a concentration of 2, 1, 0.5, 0.25, 0.125 mg/mL. The inoculum for each organism was prepared from cultures containing 10^8^ colony-forming units (CFU)/mL (MacFarland scale standard 0.5). The diluted (1:20) inoculum was applied as a drop, by means of a calibrated pipet that delivered 2 µL, resulting in a drop inoculum covering a circle of 5 mm diameter and containing 10^4^ CFU. The plates were incubated for 24 h at 37 °C. Gentamicin (250 µg/mL; Sigma) was used as positive control (Control (+)). Observations were performed by duplicate, and results are expressed as the lowest concentration of extract or fraction able to produce a complete suppression of colony growth on agar (minimum inhibitory concentration).

## 5. Conclusions

The present study allowed us to conclude that the ethanolic extract from the plant species *Acalypha arvensis* presents antibacterial activity against a couple of strains of methicillin-sensitive and methicillin-resistant *Staphylococcus aureus, Klebsiella pneumoniae,* and *Pseudomonas aeruginosa,* which supports the known medicinal applications attributed to this plant. The presence of the ellagitannin called corilagin (**1**) was isolated and identified in this species for the first time. It is one of the main constituents present in both the extract and in the active fractions (AaR4 and AaR5). Therefore, it makes sense to state that corilagin (**1**) is one of the main natural products responsible for the observed biological activity. However, we cannot discard the other phenolic-type compounds (chlorogenic acid, quercetin glycoside, caffeic acid and rutin) isolated. Therefore, it can be concluded that the aerial parts of this plant could be a good alternative in the treatment of infections caused by methicillin-resistant bacteria.

## Figures and Tables

**Table 1:** MICs of the three extracts obtained from *A. arvensis*.

Extract (mg/mL)					
Bacteria	AaHex	AaAcOEt	AaEtOH	Control (+)	Control (−)
Sa	>2	2	2	---	*
SaRM1	>2	>2	2	---	*
SaRM2	>2	>2	2	---	*
Se	>2	>2	2	---	*
Sh	>2	>2	>2	---	*
Ef	>2	>2	>2	---	*
Kp1	>2	>2	2	---	*
Kp2	>2	>2	2	---	*
Pa	>2	2	2	---	*
Ec1	>2	>2	>2	---	*
Ec2	>2	>2	>2	---	*
Ec3	>2	>2	>2	---	*
Sd	>2	>2	>2	---	*

(---): no growth; (*): growth.

**Table 2:** MIC determination of the fractions (AaR2, AaR3, AaR4, and AaR5) obtained with EtOH.

Fractions (mg/mL)						
Bacteria	AaR2	AaR3	AaR4	AaR5	Control (+)	Control (−)
Sa	>2	>2	1	0.5	---	*
SaRM1	>2	>2	<0.5	<0.5	---	*

(---): no growth; (*): growth.

## Data Availability

Data is contained within the article and supplementary material.

## Supplementary Materials

The following supporting information can be downloaded at: https://www.mdpi.com/article/10.3390/plants11030300/s1, Figure S1: Chemical structure, HPLC chromatogram and UV light spectrum of corilagin (**1**). Figure S2: ^1^H-NMR (CD_3_OD, 600 MHz) of corilagin (**1**). Figure S3: ^13^C-NMR (CD_3_OD, 150 MHz) of corilagin (**1**). Figure S4: ^13^C-DEPT-NMR (CD_3_OD, 150 MHz) of corilagin (**1**). Figure S5: ^1^H-^1^H(COSY)-NMR (CD_3_OD, 600 MHz) of corilagin (**1**). Figure S6: ^1^H-^13^C(HSQC)-NMR (CD_3_OD, 600 MHz for ^1^H and 150 MHz for ^13^C) of corilagin (**1**). Figure S7: ^1^H-^13^C(HMBC)-NMR (CD_3_OD, 600 MHz for ^1^H and 150 MHz for ^13^C) of corilagin (**1**). Figure S8: ^1^H-NMR (CD_3_OD, 600 MHz) of the mixture of compounds (**6–8**). Figure S9: ^13^C-NMR (CD_3_OD, 150 MHz) of the mixture of compounds (**6–8**). Figure S10: ^13^C-DEPT-NMR (CD_3_OD, 150 MHz) of the mixture of compounds (**6–8**). Figure S11: ^1^H-^1^H(COSY)-NMR (CD_3_OD, 600 MHz) of the mixture of compounds (**6–8**). Figure S12: ^1^H-^13^C(HSQC)-NMR (CD_3_OD, 600 MHz) of the mixture of compounds (**6–8**). Figure S13: ^1^H-^13^C(HMBC)-NMR (CD_3_OD, 600 MHz) of the mixture of compounds (**6–8**).
