# Supplementary material for: Ellagitannin, Phenols, and Flavonoids as Antibacterials from Acalypha arvensis (Euphorbiaceae)
Source: Plants (Basel). 2022 Jan 24;11(3):300. doi: 10.3390/plants11030300 (PMC8840177; doi:10.3390/plants11030300)
Supplement: Supplementary file 1 [file plants-11-00300-s001.zip › plants-1530462-supplementary.pdf]

plants

MDPI

Article

Ellagitannin, Phenols, and Flavonoids as Antibacterials from *Acalypha arvensis* (Euphorbiaceae)

Ever A. Ble-González [1,*], Abraham Gómez-Rivera [1], Alejandro Zamilpa [2], Ricardo López-Rodríguez [1], Carlos Ernesto Lobato-García [1], Patricia Álvarez-Fitz [3], Ana Silvia Gutierrez-Roman [2], Ma Dolores Perez-García [2], Alejandro Bugarin [4] and Manasés González-Cortazar [2,*]

1 División Académica de Ciencias Básicas, Universidad Juárez Autónoma de Tabasco, Carretera Cunduacán-Jalpa Km. 0.5, Cunduacán Tabasco 86690, Mexico; abgori@gmail.com (A.G.-R.); ricardo.lopezr@ujat.mx (R.L.-R.); carlos.lobato@ujat.mx (C.E.L.-G.)
2 Centro de Investigación Biomédica del Sur, Instituto Mexicano del Seguro Social, Argentina No. 1, Col. Centro, Xochitepec, Morelos 62790, Mexico; azamilpa_2000@yahoo.com.mx (A.Z.); 12gtr.ana@gmail.com (A.S.G.-R.); lola_as@yahoo.com.mx (M.D.P.-G.)
3 Cátedra CONACyT-Universidad Autónoma de Guerrero, Av. Lázaro Cárdenas s/n. Col. La Haciendita, Chilpancingo, Guerrero 39070, Mexico; paty_fitz@hotmail.com
4 Department of Chemistry and Physics, Florida Gulf Coast University, Fort Myers, FL 33965, USA; abugarin@fgcu.edu
* Correspondence: ble_49@hotmail.com (E.A.B.-G.); gmanases2000@gmail.com (M.G.-C.); Tel.: +52-(993)-581-500 (ext. 4711) (E.A.B.-G.); +52-(777)-3612-155 (M.G.-C.)

Keywords: *Acalypha arvensis*; ellagitannin; corilagin; *Staphylococcus aureus*; flavonoids; antibacterial

Citation: Ble-González, E.A.; Gómez-Rivera, A.; Zamilpa, A.; López-Rodríguez, R.; Lobato-García, C.E.; Álvarez-Fitz, P.; Gutierrez-Roman, A.S.; Perez-García, M.D.; Bugarin, A.; González-Cortazar, M. Ellagitannin, Phenols, and Flavonoids as Antibacterials from *Acalypha arvensis* (Euphorbiaceae). *Plants* **2022**, *11*, 300. https://doi.org/10.3390/plants11030300

Academic Editors: Juei-Tang Cheng, I-Min Liu and Szu-Chuan Shen

Received: 13 December 2021
Accepted: 13 January 2022
Published: 24 January 2022

Publisher's Note: MDPI stays neutral with regard to jurisdictional claims in published maps and institu-tional affiliations.

Copyright: © 2022 by the authors. Submitted for possible open access publication under the terms and conditions of the Creative Commons Attribution (CC BY) license (http://creativecommons.org/licenses/by/4.0/).

Contents

Figure caption.

Figure S1. Chemical structure, HPLC chromatogram and UV light spectrum of corilagin (**1**)

Figure S2. ^{1}H-NMR (CD_3OD, 600 MHz) of corilagin (**1**).

Figure S3. ^{13}C-NMR (CD_3OD, 150 MHz) of corilagin (**1**)

Figure S4. ^{13}C-DEPT-NMR (CD_3OD, 150 MHz) of corilagin (**1**)

Figure S5. ^{1}H-^{1}H(COSY)-NMR (CD_3OD, 600 MHz) of corilagin (**1**)

Figure S6. ^{1}H-^{13}C(HSQC)-NMR (CD_3OD, 600 MHz for ^{1}H and 150 MHz for ^{13}C) of corilagin (**1**)

Figure S7. ^{1}H-^{13}C(HMBC)-NMR (CD_3OD , 600 MHz for ^{1}H and 150 MHz for ^{13}C) of corilagin (**1**)

Figure S8. ^{1}H-NMR (CD_3OD, 600 MHz) of the mixture of compounds (**6**-**8**).

Figure S9. ^{13}C-NMR (CD_3OD, 150 MHz) of the mixture of compounds (**6**-**8**).

Figure S10. ^{13}C-DEPT-NMR (CD_3OD, 150 MHz) of the mixture of compounds (**6**-**8**).

Figure S11. ^{1}H-^{1}H(COSY)-NMR (CD_3OD, 600 MHz) of the mixture of compounds (**6**-**8**).

Figure S12. ^{1}H-^{13}C(HSQC)-NMR (CD_3OD, 600 MHz) of the mixture of compounds (**6**-**8**).

Figure S13. ^{1}H-^{13}C(HMBC)-NMR (CD_3OD, 600 MHz) of the mixture of compounds (**6**-**8**).

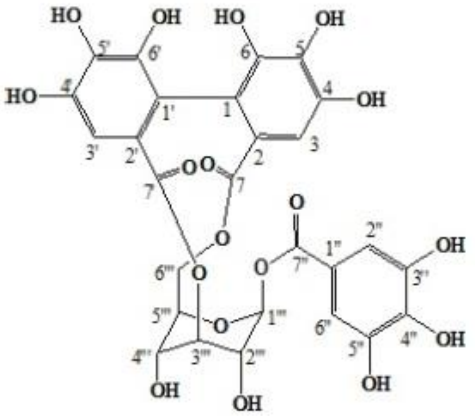

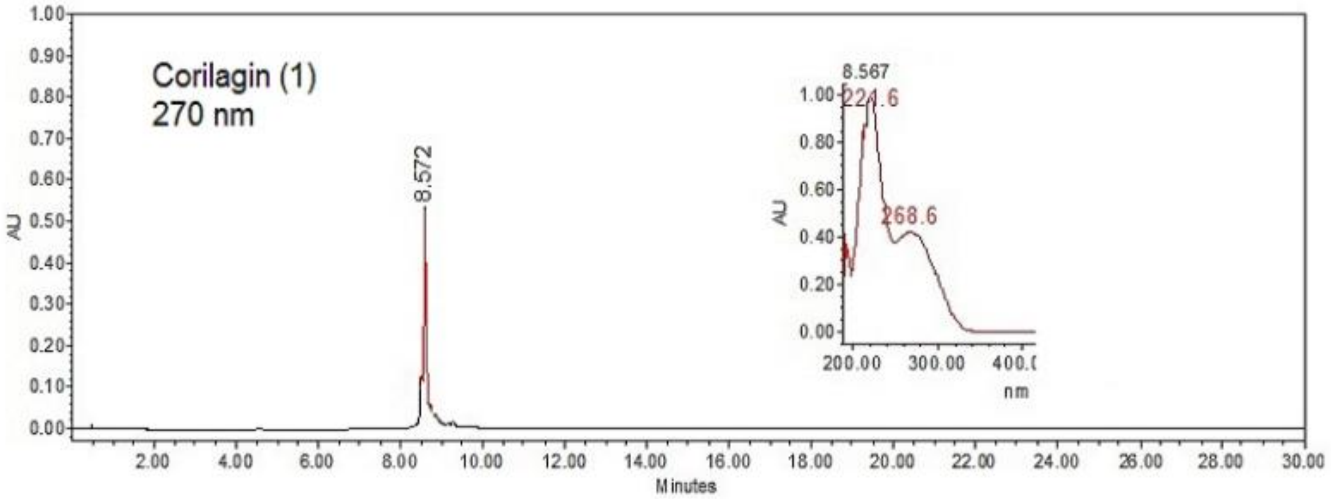

Figure S1. Chemical structure, HPLC chromatogram and UV light spectrum of corilagin (**1**).

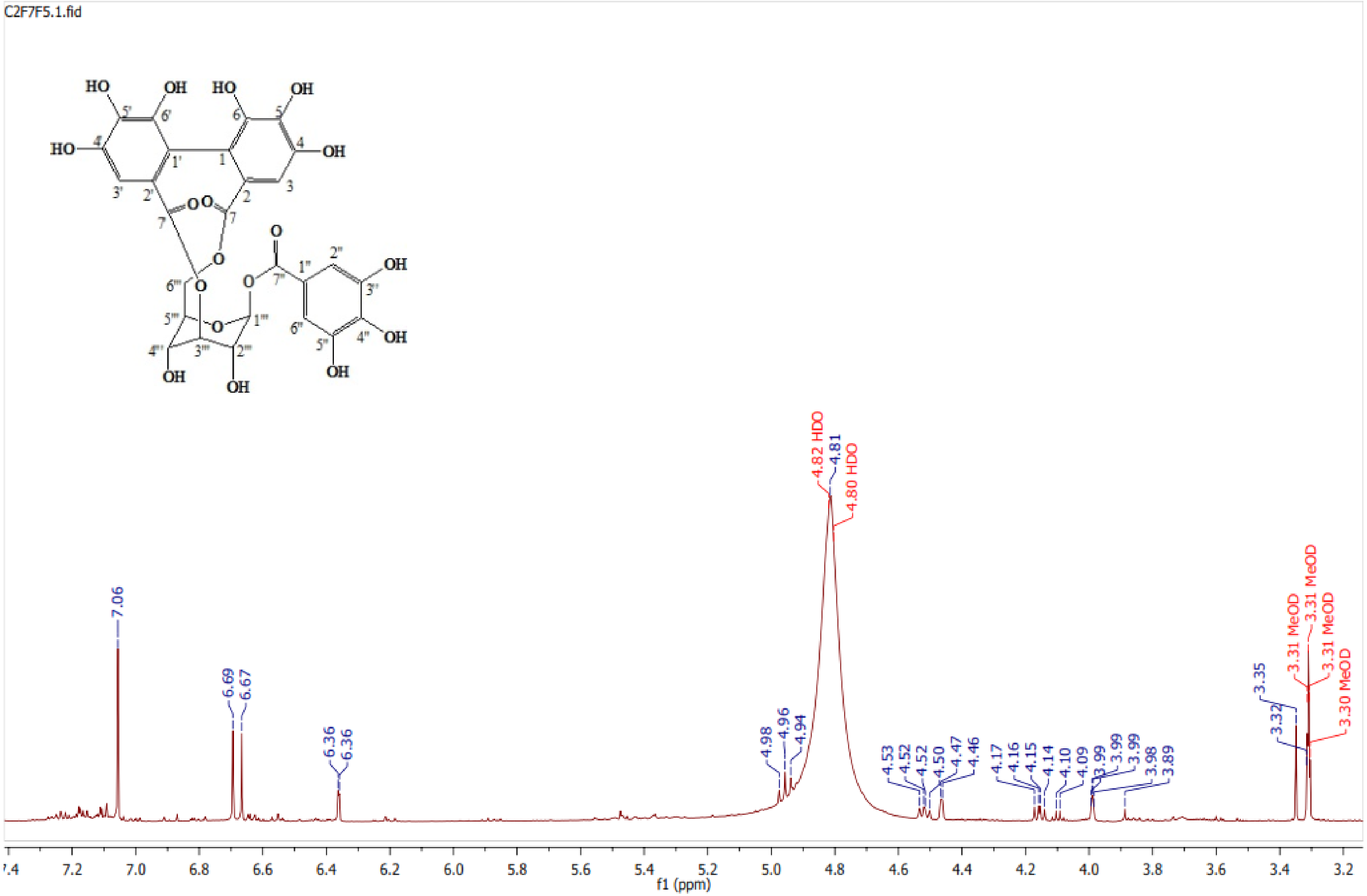

Figure S2. ^{1}H-NMR (CD_3OD, 600 MHz) of corilagin (**1**).

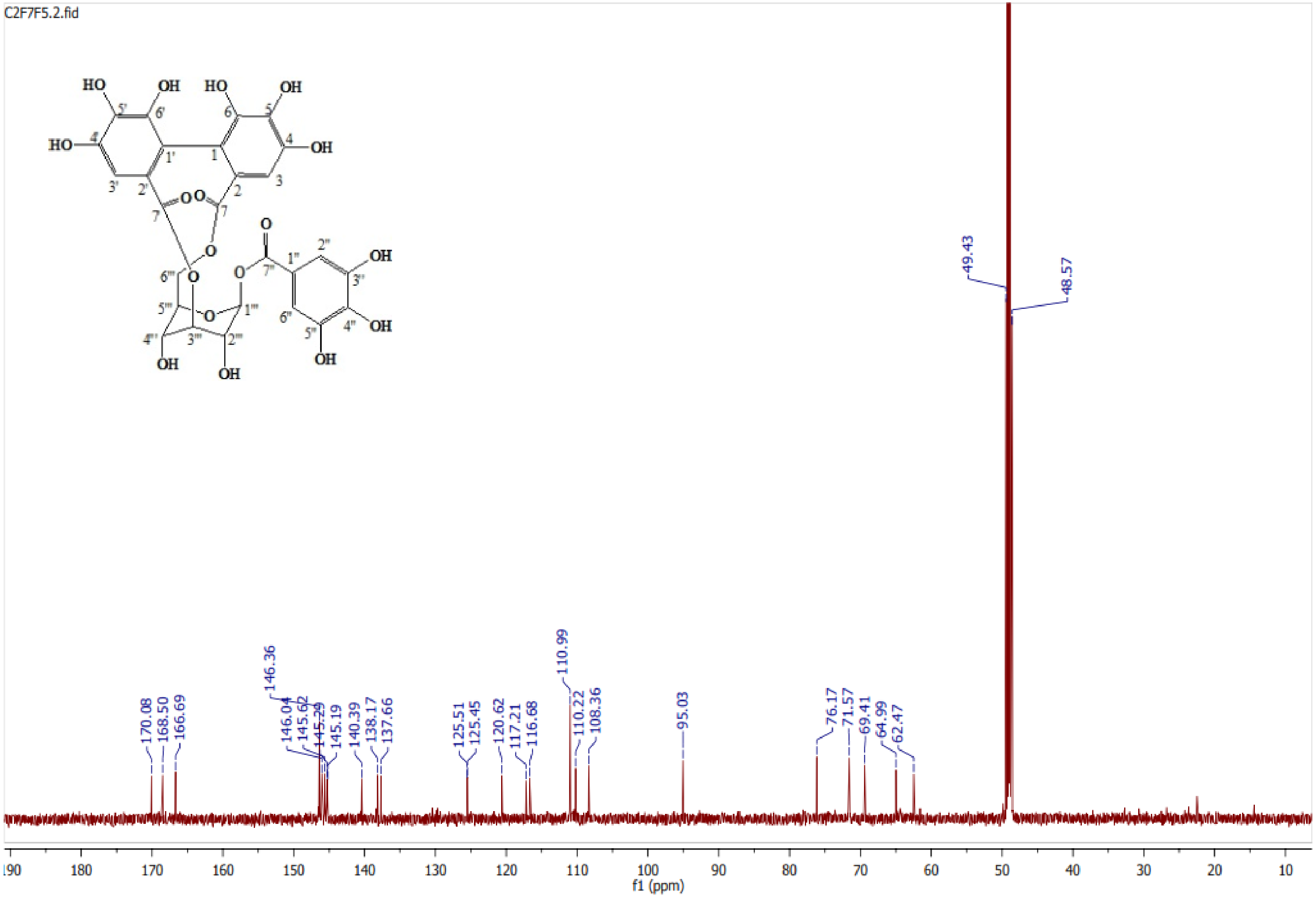

Figure S3. ^{13}C-NMR (CD_3OD, 150 MHz) of corilagin (**1**).

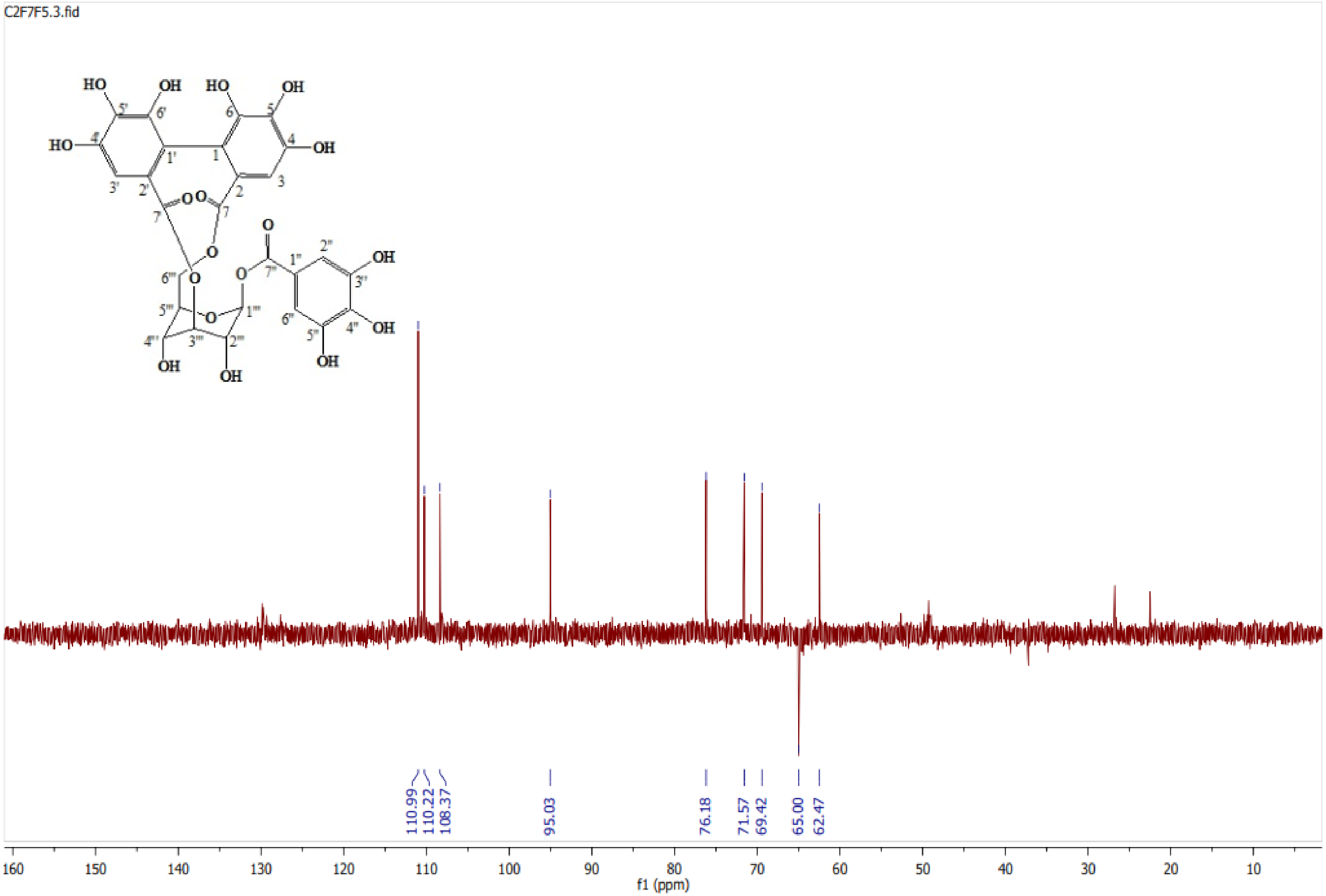

Figure S4. ^{13}C-DEPT NMR (CD_3OD,150 MHz) of corilagin (**1**)

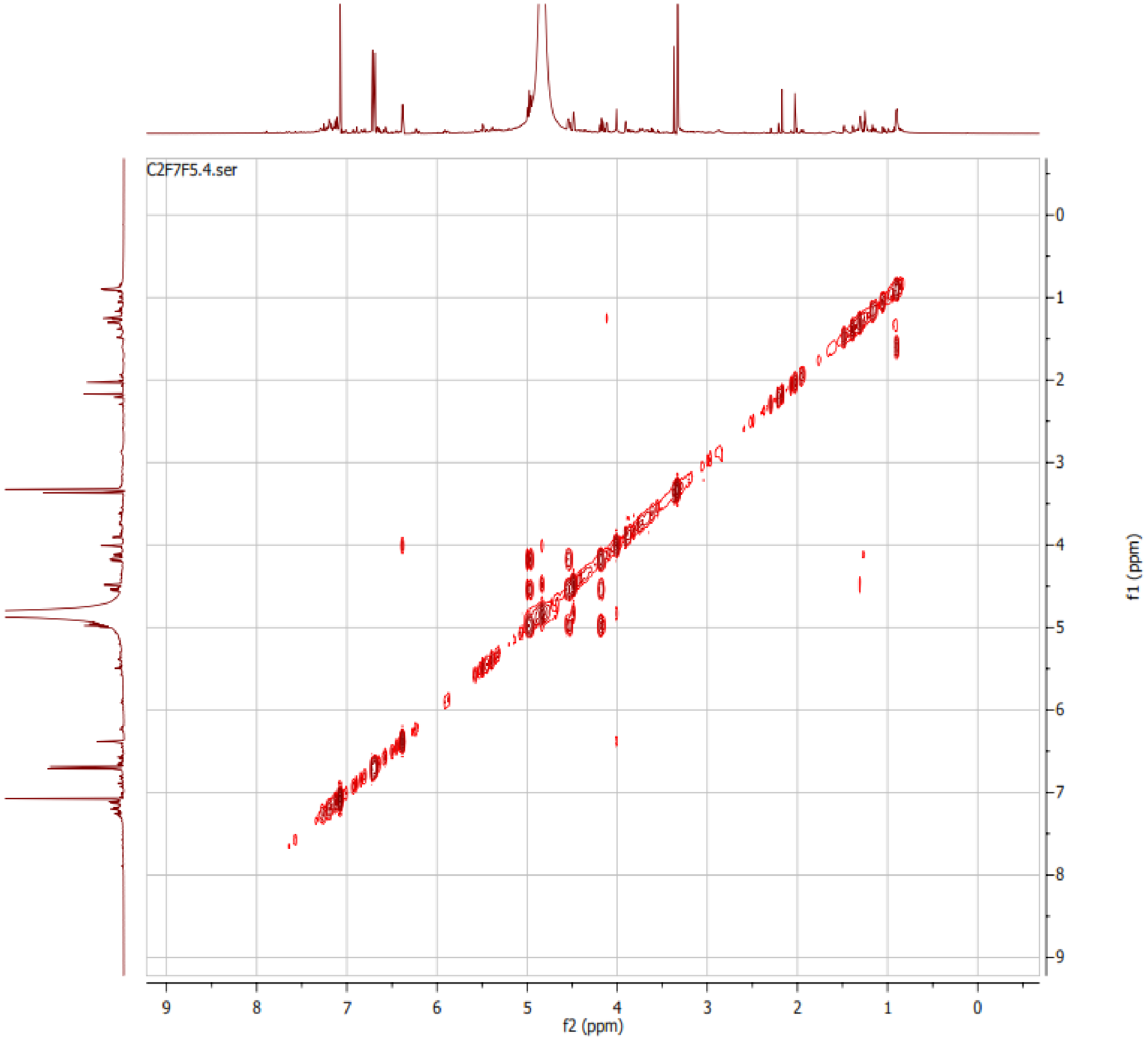

Figure S5. ^{1}H-^{1}H (COSY) NMR (CD_3OD, 600 MHz) of corilagin (**1**)

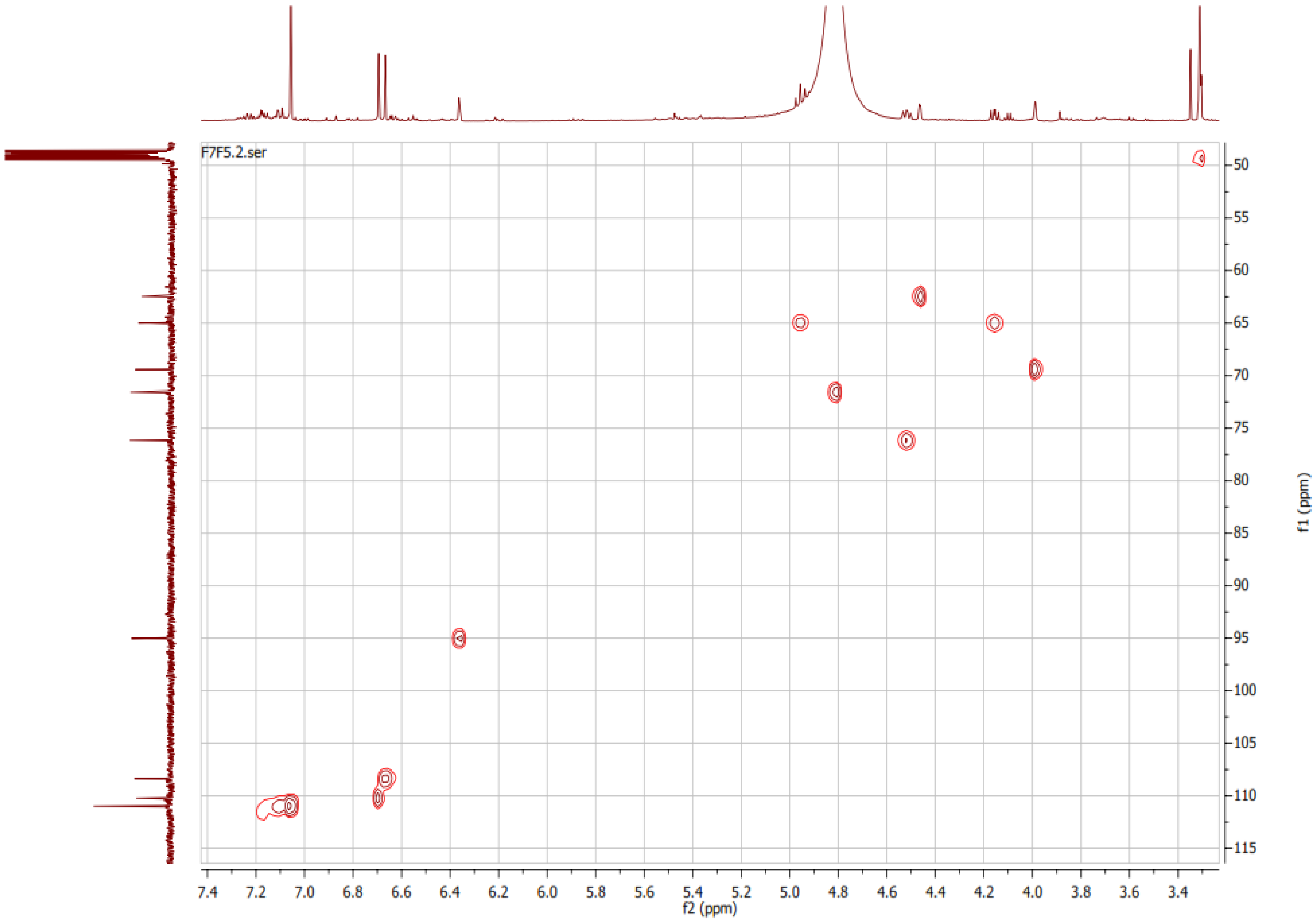

Figure S6. ^1H-^{13}C (HSQC) NMR (CD_3OD, 600 MHz) of corilagin (**1**)

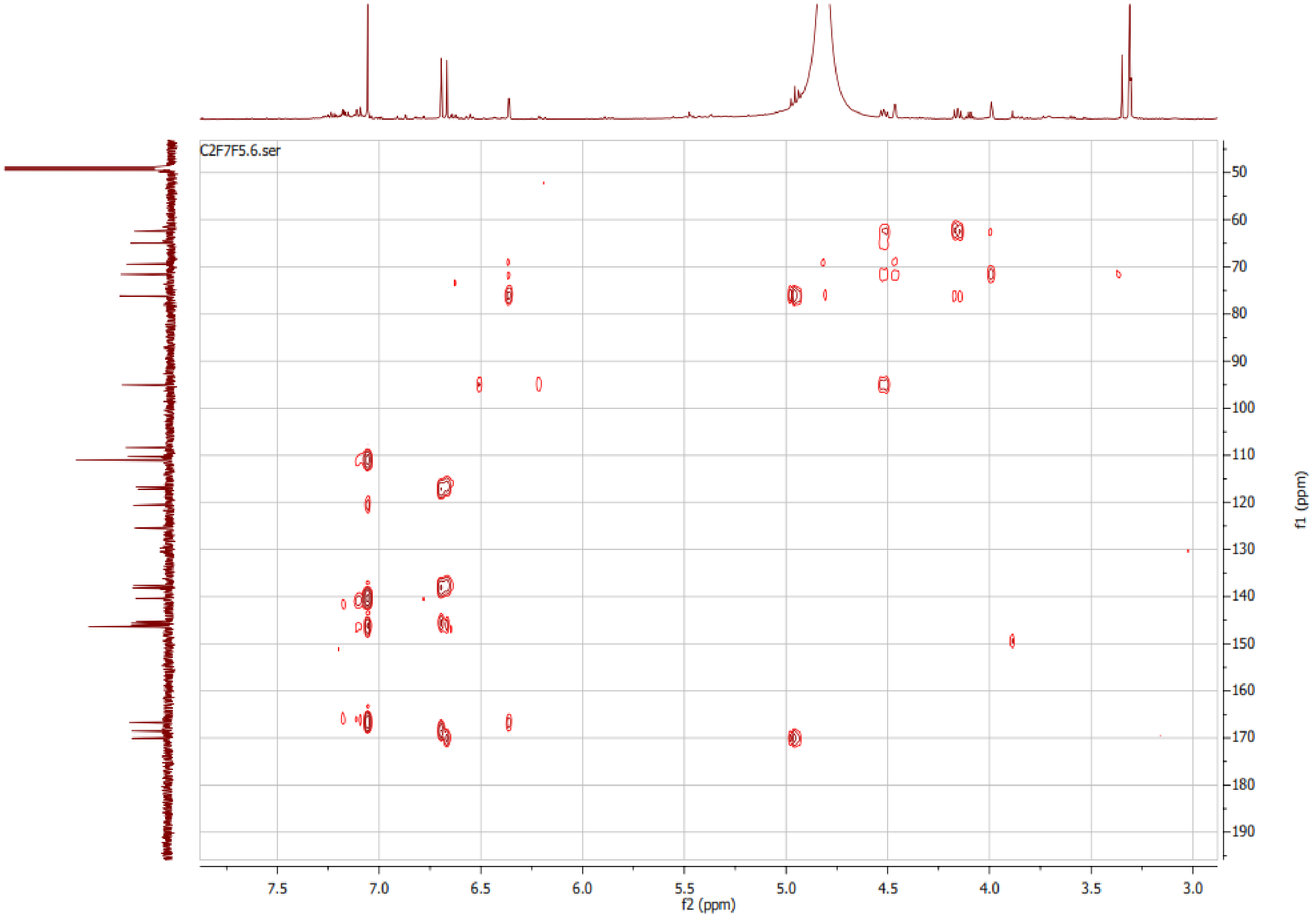

Figure S7. ^1H-^{13}C (HMBC) NMR (CD_3OD, 600 MHz) of corilagin (**1**)

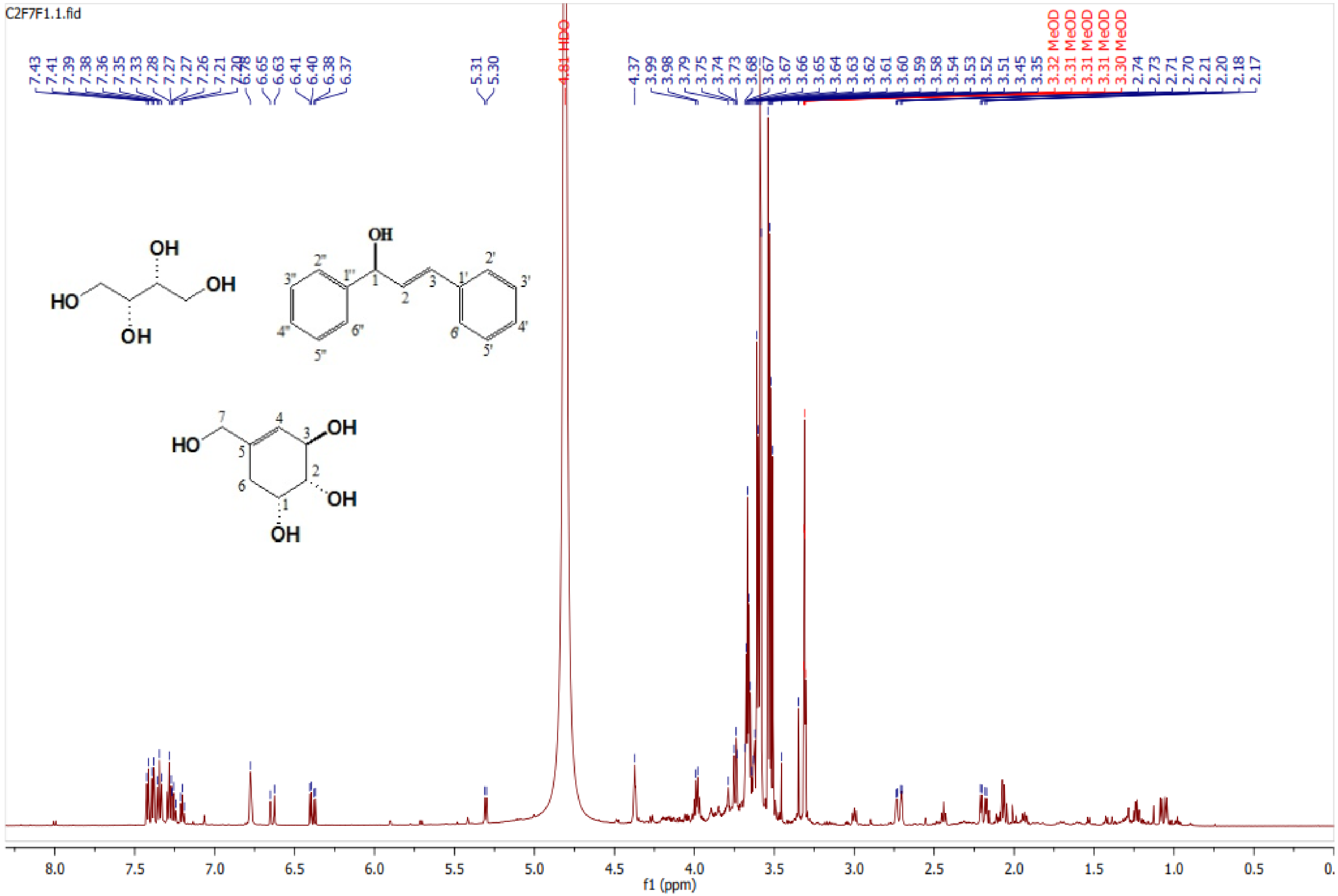

Figure S8. ^{1}H-NMR (CD_3OD, 600 MHz) of the mixture of compounds (**6-8**).

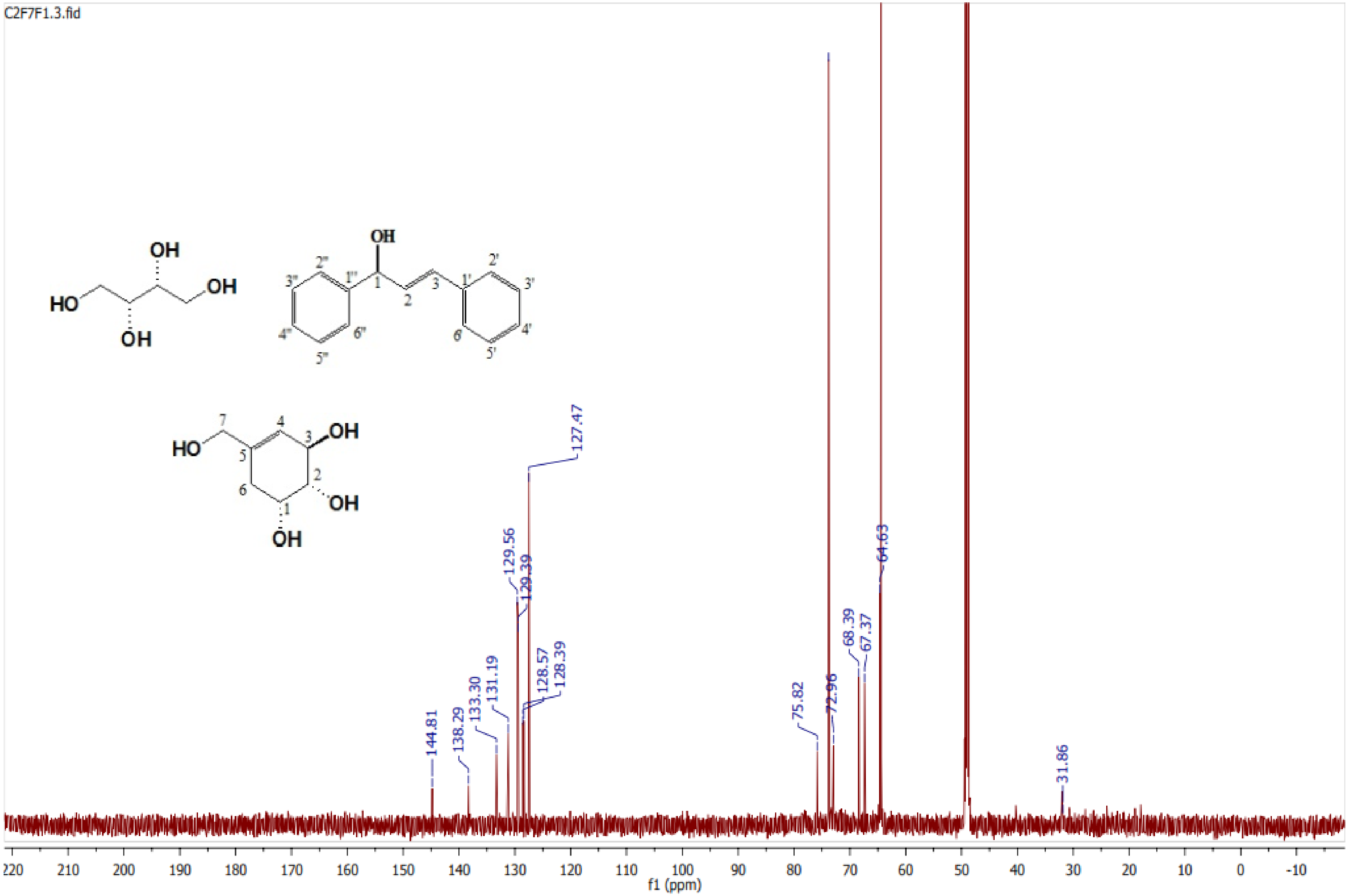

Figure S9. ^{13}C-NMR (CD_3OD, 150 MHz) of the mixture of compounds (**6-8**).

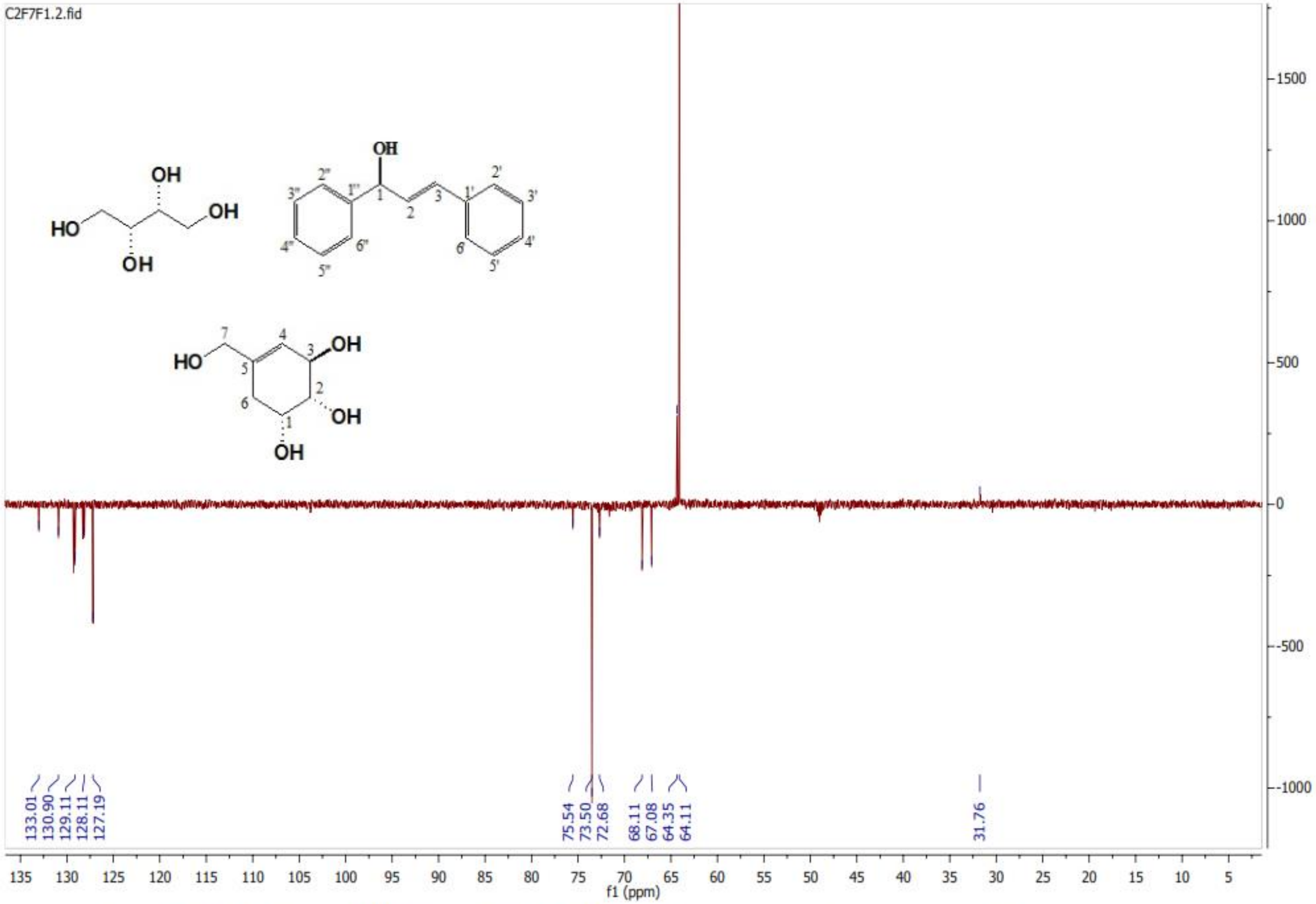

Figure S10. ^{13}C-DEPT NMR (CD_3OD, 150 MHz) of the mixture of compounds (**6-8**).

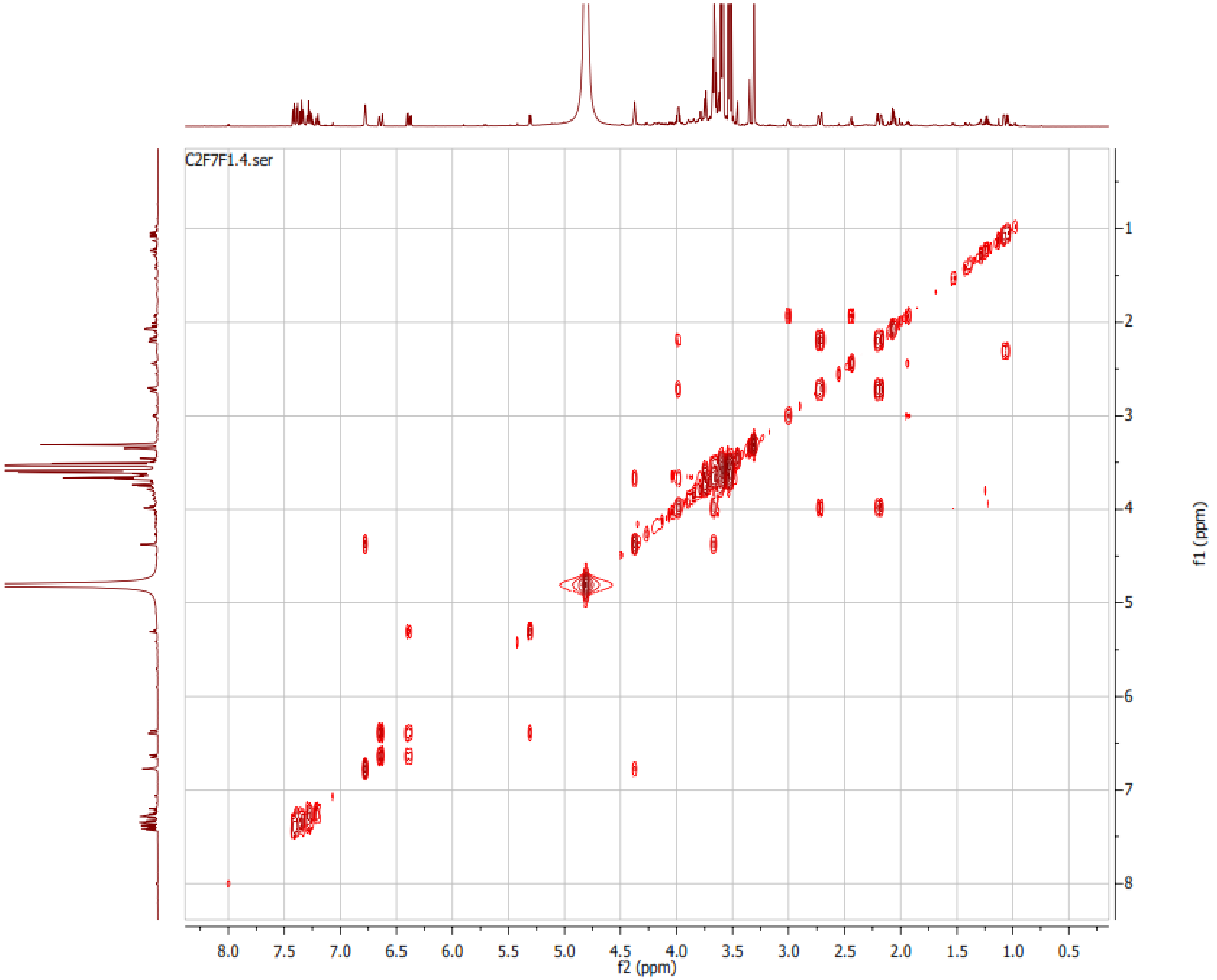

Figure S11. ^{1}H-^{1}H (COSY) NMR (CD_3OD, 600 MHz) of the mixture of compounds (**6-8**).

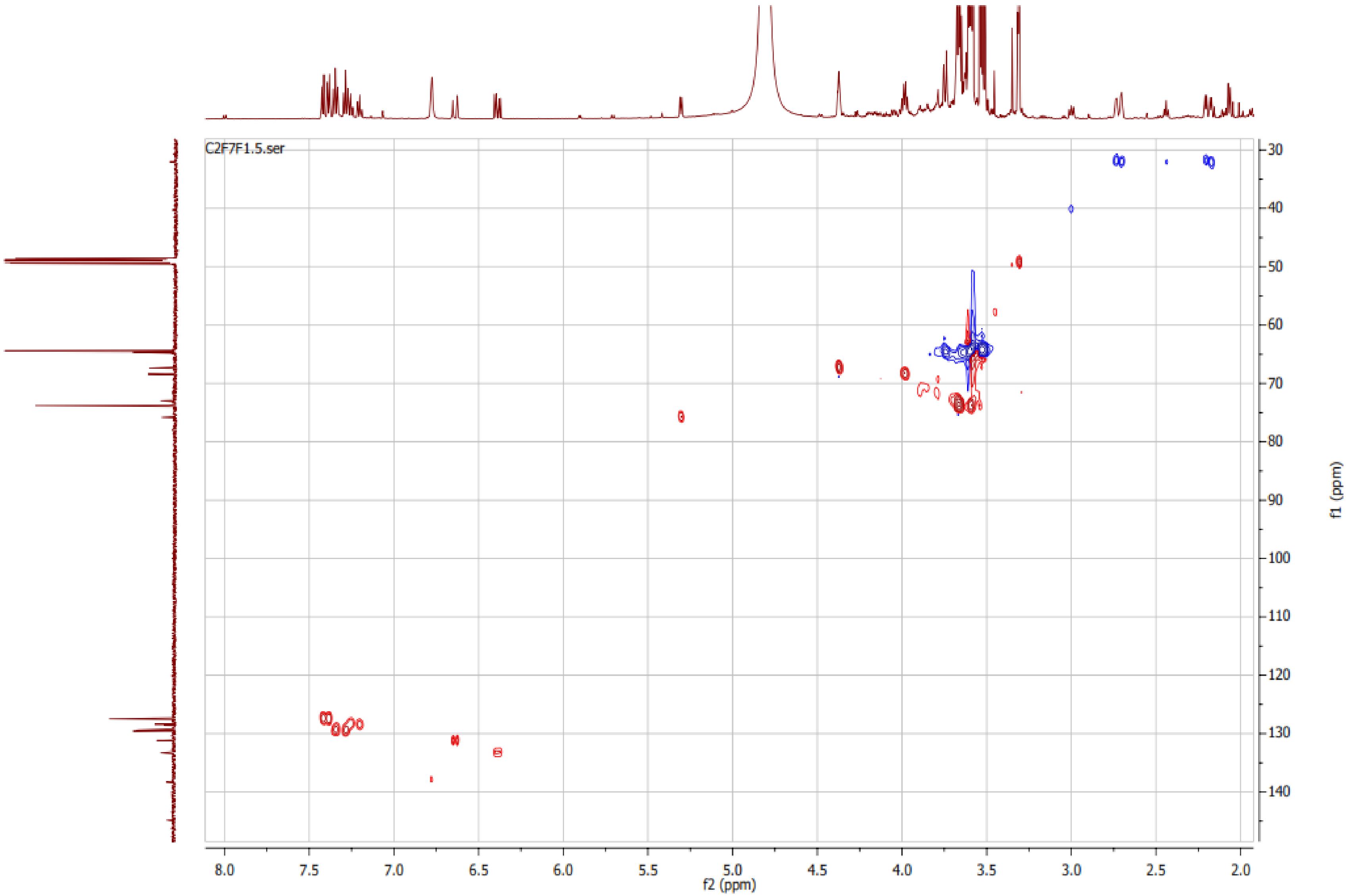

Figure S12. ^{1}H-^{13}C (HSQC) NMR (CD_3OD, 600 MHz) of the mixture of compounds (**6-8**).

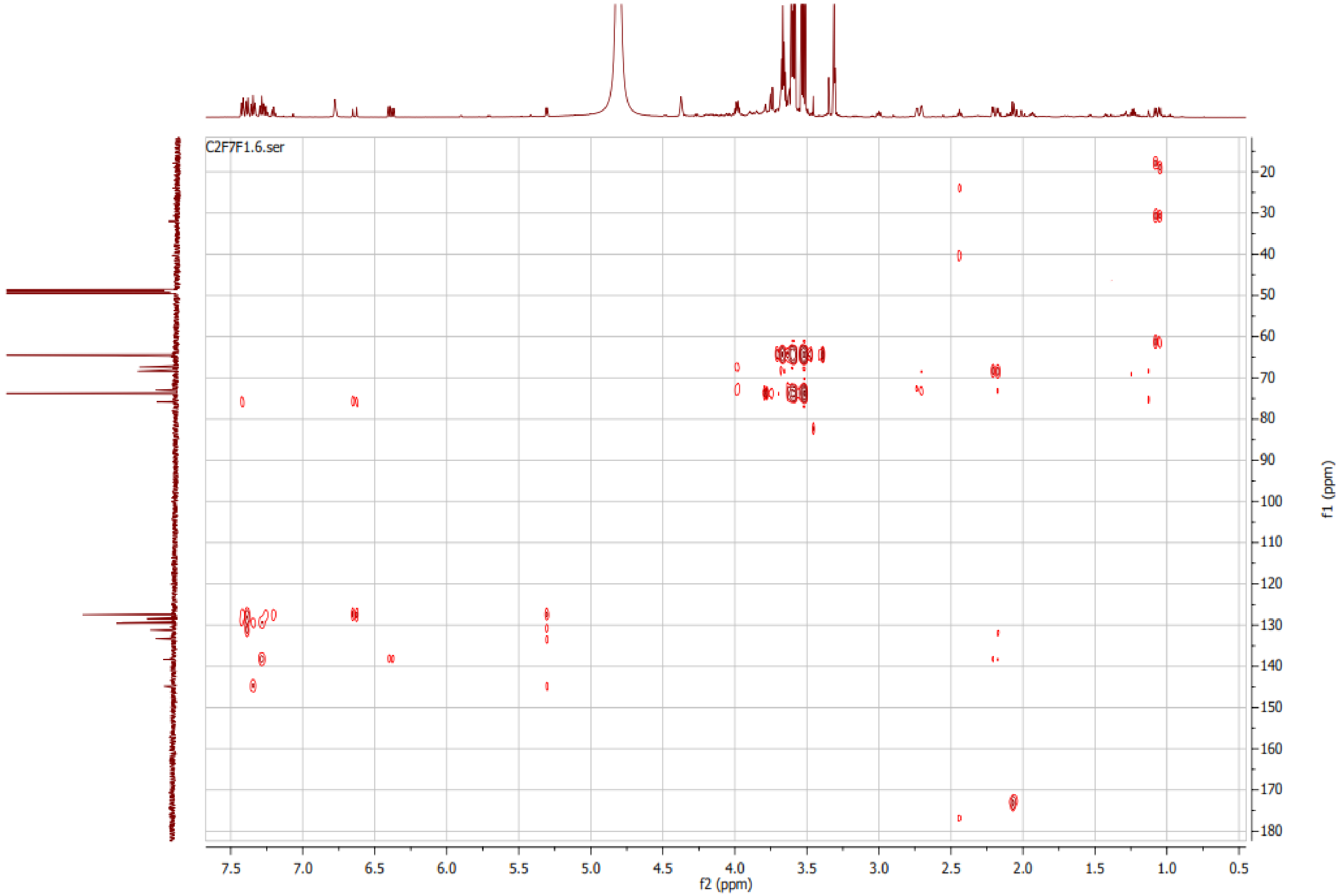

Figure S13. ^{1}H-^{13}C (HMBC) NMR (CD_3OD, 600 MHz) of the mixture of compounds (**6-8**).
